# Child and adolescent psychiatry training in Nepal: early career psychiatrists’ perspective

**DOI:** 10.1186/s13034-020-00319-5

**Published:** 2020-04-06

**Authors:** Utkarsh Karki, Yugesh Rai, Gunjan Dhonju, Eesha Sharma, Preeti Jacob, John Vijay Sagar Kommu, Shekhar P. Seshadri

**Affiliations:** 1 Department of Child and Adolescent Psychiatry, National Institute of Mental Health and Neuro Sciences, Bengaluru, India; 2 Essex Partnership University NHS Trust, Colchester, UK; 3 Child and Adolescent Psychiatry Unit, Kanti Children’s Hospital, Kathmandu, Nepal

**Keywords:** Child and adolescent psychiatry, Training, Early career psychiatrists, Nepal, Low income country

## Abstract

**Background:**

Nepal is a developing low-income country in Southeast Asia. There is a huge burden of child and adolescent mental health (CAMH) in Nepal which has a population of around 29 million and 40–50% of the population comprises of children and adolescents. Child and Adolescent Psychiatry (CAP) has not been formally recognized as a subspecialty in Nepal and there is no standardized curriculum for CAP training. The objectives of the survey were to identify the current status of training, shortfalls and to explore the training experiences of early career psychiatrists (ECPs) in Nepal.

**Methods:**

The participants were ECPs in Nepal. An online questionnaire was created consisting of 20 questions including 3 questions requiring an answer indicating a level of agreement scored on a ten-point scale. Questionnaire using google form was e-mailed with the uniform resource locator (URL). Respondents anonymously answered the questions. The survey was open from 01/01/2019 to 01/04/2019.

**Results:**

Response rate was 83.69%. Around 42% (n = 32) were trainees in Doctor of Medicine (MD) Psychiatry and 58% (n = 45) of respondents had completed their MD Psychiatry. More than half of the ECPs had not received formal training specific to CAP. Seventy percent (n = 54) ECPs reported that their current workplace did not have a specific unit to address psychological problems in children and adolescents. However, 62% (n = 48) of ECPs came across 10 CAP cases per week. On a ten-point scale, mean score of ECPs confidence in diagnosing, management and overall confidence in CAP cases were 5.18 ± 1.56, 4.58 ± 1.59 and 4.67 ± 1.62 respectively. Fifty-four percent (n = 42) of respondents rated their training as limited and 74% (n = 57) of them wanted additional training in CAP. Psychological intervention, psychotherapy and a fellowship course were the additional training most of the ECPs wanted to receive.

**Conclusion:**

Despite significant exposure to CAP patients in daily practice, ECPs self-evaluated their training as inadequate and there is no standardized CAP training program in Nepal for ECPs. The desire of ECPs to receive additional training in CAP is highly encouraging and positive. We advocate for the development and incorporation of CAP training in current psychiatry training to fulfill these unmet training needs in Nepal.

## Background

Children and adolescents constitute almost a third (2·2 billion individuals) of the world’s population and almost 90% live in low and middle-income countries (LAMIC), where they form up to 50% of the population. Mental health problems affect 10–20% of children and adolescents worldwide [1].

Half of the mental disorders are estimated to have onset by the age of 14 years [2]. Early identification and treatment of these disorders can improve the outcome and prognosis of psychiatric illnesses. However the major hindrances for mental health of children and adolescents in LAMIC are-lack of evidence on delivery of treatments, low levels of detection of child mental disorders, and shortage of skilled child mental health professionals [3]. Therefore, in these countries there is a vast gap between child and adolescent mental health (CAMH) needs (as measured through burden of disease estimates) and the availability of CAMH resources. The term “early career psychiatrists’” include psychiatry trainees and psychiatrists within 7 years after specializing in psychiatry (i.e. finishing the whole time equivalent postgraduate specialty training in the respective country. [4].

Nepal is a low-income country in Southeast Asia. It has a population of around 29 million, and 40–50% of the population comprise of children and adolescents [5]. The Ministry of Health and Population of Nepal estimates that about 15–20% of this population (2–3 million) may suffer from some form of mental disorder [6, 7]. Many children in Nepal are living under multidimensional poverty as measured across health, education and living standards [8]. They are exposed to child labor, war, violence, sexual abuse, human trafficking and natural disasters like earthquakes, floods and landslides. Such factors are shown to have negative influences on child development ultimately leading to mental illnesses [9].

According to the World Health Organization Assessment Instrument for Mental Health Systems (WHO–AIMS) report on mental health in Nepal 2006, there were 18 outpatient mental health facilities available, 3-day treatment facilities, and 17 community based inpatient psychiatry facilities in addition to the mental hospital in Kathmandu, but there were no separate facilities for children and adolescents [10]. Only recently, has there been greater importance given to identifying and treating mental disorders in children [6, 7]. The only child and adolescent psychiatry service in Nepal was set up at Kanti Children’s Hospital, Kathmandu in collaboration with Non-Governmental Organizations (NGOs) in July 2015; there are as yet no inpatient facilities [11]. There is also an acute shortage of child and adolescent mental health professionals with only two child and adolescent psychiatrists in the whole country.

Currently, about 20 Medical Colleges are operating in Nepal with a minimum time period for completion of Bachelor of Medicine and Surgery (MBBS) course between 5 and 1/2 years to 6 years. Post graduate training (residency) or MD courses are run in 14 out of 20 medical colleges. However, training for MD Psychiatry are provided in only 12 out of the 14 medical colleges. Child and Adolescent Psychiatry services and training provisions are very limited and confined to only few medical colleges. No specialized post graduate training in child and adolescent psychiatry is available in Nepal. Whether educators provide training of sufficient duration and depth for trainees to assess, diagnose and manage child and adolescent psychiatry patients remains unevaluated.

Taken together, despite the large number of child and adolescent mental illnesses and the importance for early treatment, there is no information available about child and adolescent psychiatry training in Nepal. The current study was designed to understand whether the current psychiatric training, duration of training and modality of training is adequate for trainees to assess, diagnose and manage children and adolescents with mental disorders. Understanding of the current status of CAP training across Nepal, short falls and training needs can serve to improve standards of training and sensitize the concerned authorities to develop and incorporate CAP training curriculum in the post graduate training in Nepal as well as other LAMIC.

## Methods

### Participants

The participants of this study were early career psychiatrists in Nepal. Early career psychiatrists (psychiatry trainees and psychiatrists within 7 years after specializing in psychiatry) were contacted through email and a questionnaire to be filled via google form was sent with the URL. The Psychiatric Association of Nepal (PAN) was contacted to collect the list of early career psychiatrists. Representatives or members of PAN from their respective home towns were contacted via e mail to obtain the contacts (email) of individuals who are currently undergoing training in Psychiatry. The study’s objective was clearly stated on the online survey system’s web page. The survey was conducted anonymously. A consent form was sent along with the URL link. Answering the questionnaire after reading the consent was considered to constitute consent to participate in the study. Ethics approval was sought from the Ethics Committee of National Institute of Mental Health and Neuro Sciences (NIMHANS), Bengaluru. The survey period was from 1st January to 1st April 2019.

### Questionnaire

The questionnaire consisted of twenty questions which included: (1) description of the respondents (2) CAP training characteristics (3) CAP self -evaluation and (4) three open ended questions regarding their desire for additional CAP training.

The survey contained three types of responses: single and multiple-choice responses, open responses and responses on a ten -point Likert scale.

### Statistical analysis

Study results were expressed as frequency, percentages and mean ± SD.

## Results

**Fig. 1:**
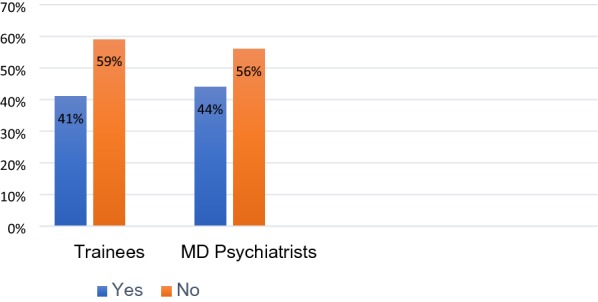
Did ECP’s receive training specific to CAP?

Out of 92 ECPs we received 77 (83.69%) responses. Tables 1, 2 and Fig. 1 summarizes the description of ECPs and their training characteristics. Table 3 summarizes CAP self-evaluation and perceived training needs.

**Table 1:** Description of the early career psychiatrists’ (n = 77)

Characteristics		n (%)
Current positions		
Trainee in MD psychiatry		
Year 1		12 (37.5)
Year 2		10 (31.3)
Year 3		10 (31.3)
Total		32 (41.6)
Completed MD psychiatry		
1 year		11 (24.4)
2 years		9 (20.0)
3 years		14 (31.1)
4 years		6 (13.3)
5 years		5 (11.1)
Total		45 (58.4)

**Table 2:** Description of training characteristics

Characteristics	n (%)
Type of CAP training receiving or received	
E-learning/online training	7 (9.3)
Clinical postings	38 (50.7)
Group discussions	28 (37.3)
Case presentations	47 (62.7)
Minimal or no formal training	25 (33.3)
Dedicated CAP unit or department in the place of training or work	
Present	23 (29.9)
Absent	54 (70.1)
No of CAP patients seen per week	
None	3 (3.9)
One to ten	48 (62.3)
Ten to twenty	19 (24.7)
More than twenty	7 (9.1)
How often are childhood psychiatric disorders suspected	
Always suspected	5 (6.5)
Often suspected	51 (66.2)
Sometimes suspected	21 (27.3)

**Table 3:** CAP self-evaluation and perceived training needs(n = 77)

Characteristics	n (%)
Overall rating in CAP training	
Comprehensive	1 (1.3)
Satisfactory	22 (28.6)
Limited	42 (54.5)
Grossly inadequate	12 (15.6)
Readiness to take on a consultant role in CAP	
Ready for the role	5 (6.5)
Adequate but would benefit from further training	38 (49.4)
Unprepared	34 (44.2)
Want additional training in CAP	
Yes	57 (74)
No	4 (5.3)
May be	16 (20.8)
Mental health to be included in the curriculum for children and adolescents	
Extremely important	74 (96.1)
Slightly important	3 (3.9)
Confidence in diagnosing CAP cases^a^	5.18 ± 1.56
Confidence in management/intervention of CAP cases^a^	4.58 ± 1.59
Overall confidence in CAP^a^	4.67 ± 1.62

^a^ These results (score on a ten-point scale with 10 being the highest possible score) are expressed as the mean ± SD

Answers for the open-ended questions regarding their desire of additional CAP training, the majority have mentioned about wanting to learn psychological interventions, psychotherapy and a desire to undergo a short-term fellowship course in CAP. In addition, they have mentioned about the need to increase awareness, advocacy, conducting regular continued medical education (CME) on CAMH and incorporating CAP in their MD psychiatry training.

## Discussion

This is the first study to explore the training experiences of ECPs of Nepal in Child and Adolescent Psychiatry. Our study reveals that about half of the ECPs have not received formal training specific to CAP. Although CAP has been incorporated in postgraduate training curriculum, there exists large variation in service organization, clinical exposure and supervision. CAP is not recognized as a subspecialty and currently lacks an international standardized training program [12] and the majority of trainees receive supervision from senior general psychiatrists. The post graduate training is often aligned with adult psychiatry and more weightage is given to general adult psychiatry in clinical examination. A specialized post graduate training in CAP is non-existent in countries like Nepal and Vietnam [12]. In the Far East, there is an overall underdevelopment of CAP post graduate training systems with paucity of official guideline for CAP training [13]. On a positive note, India a neighboring country to Nepal has around 6 institutes for specialty training in child and adolescent psychiatry [14].

Seventy percent of ECPs reported that they did not have any specific unit or department to address psychological problems in children and adolescents. Most of CAP patients are reviewed in general psychiatric clinic and only few places have specific child guidance clinic. There are no specific inpatient facilities for child and adolescent and unfortunately children get admitted in adult psychiatric units. Furthermore, 62% of ECPs would come in clinical contact with 10 CAP cases per week during their training or clinical practice. Commonly seen disorders by ECPs during their training or current practice were neurodevelopmental disorders followed by dissociative disorders and anxiety disorders. It is reported that psychiatric trainees will often experience insufficient CAP cases in their general psychiatry training and senior psychiatrists might need to supervise trainees without enough CAP experience in their own training or practice [15]. There has not been an increase in CAP workforce despite an increase in the total number of psychiatrists in Nepal. With only two child psychiatrists in Nepal, there is an urgent need to train ECPs in CAP to meet the current service demands of mental health problem in children. It is encouraging to find a few post-graduate training centers have started to send trainees to Kanti Children Hospital, Nepal and NIMHANS, India for CAP exposure.

Several studies [16] have shown that exposure to certain subspecialties of psychiatry during initial phase of postgraduate training could have positive impact on career choice. In Nepal, the ratio of child and adolescent psychiatrists for 100,000 children aged 14 years or younger is 0.01 [12]. CAP can be made an attractive career choice by exposure to successfully treated cases, provision of educational seminars, case conferences, and a well-organized multidisciplinary team for CAP practice. Our study showed that half of the ECPs had clinical posting in CAP and engaged in case presentations. It is discouraging to find that one-third had no or minimal exposure to CAP in their residency training. We speculate that this figure might be higher than reported in this study as some ECPs were trained in Indian institutes that provide CAP exposure. In addition, two-third of ECPs rated their training in CAP as limited to grossly inadequate which needs to be addressed in the coming days but 22.6% of ECPs were satisfied with their training in CAP. It would have been prudent to explore the reasons for satisfaction, but we failed to capture this with the devised questionnaire. We speculate that those ECPs trained in few institutes with CAP training in Nepal and institutes in India may have constituted the 22.6% of ECPs satisfied with training. A study conducted in India with 451 respondents who had undergone post-graduate training in psychiatry perceived a rotation in CAP as a necessity and in the same study it was found that 26.2% of the group perceived the quality of their training in CAP to be “poor” or “very poor” [17]. This percentage is very less compared to our study wherein two-third of the ECPs perceived the training in CAP as limited or grossly inadequate. This could be due to the reason that post-graduate curricula for psychiatry in India stipulate mandatory postings in CAP for 2 or 3 months [18, 19].

Despite the average to below average self-rating in their confidence in diagnosing and management of CAP case (5.18 ± 1.56 and 4.58 ± 1.59) it is heartening and encouraging to know that two-third (74%) of ECPs want additional training in CAP. A similar survey done in Japan demonstrated that early career psychiatrists’ self-evaluated their clinical CAP experience as insufficient, and their CAP experience and CAP interest were found to correlate significantly [20]. Ninety-six percent (n = 74) also identify the importance of mental health in children and suggest it to be included in the curriculum of children and adolescents. However, obstacles to development of CAP postgraduate training may be stigma towards mental health issues and lack of funding which needs to be overcome.

With the above findings of the present study, we would like to emphasize the need of exposure of early career psychiatrists to more CAP cases to facilitate adequate and successful recruitment into CAP. In this regard, we recommend that all psychiatric training programs require (1) at least 2 months of intensive CAP training during residency under the supervision of a qualified child and adolescent psychiatrist, (2) short term training courses/continued medical education/workshops on specific topics to enhance the psychiatric trainees’ clinical skills to diagnose and treat child and adolescent cases.

## Limitations

The answers to the survey were subjective assessments by ECPs and the respondent’s training and clinical experience and diagnostic/treatment skills were not assessed objectively. Also, information was not obtained directly from the training program in each institution and the intention of each question could be interpreted slightly differently by respondents. Lastly, more qualitative exploration would have provided us with a greater understanding of CAP training and its shortfalls.

## Conclusion

Our survey results show that despite significant exposure to CAP patients in daily practice, there is no standardized CAP training program in Nepal. Also, ECPs self-evaluated their training in CAP as insufficient. The desire of ECPs to receive additional training in CAP is highly encouraging and positive. In order to develop interest in CAP, more young psychiatrists need to be provided more exposure to CAP cases during the early stages of psychiatric training. We advocate for the development and incorporation of competency-based CAP training program in current post-graduate psychiatry training to fulfill these unmet training needs in Nepal.

## Abbreviations

CAP: Child and Adolescent Psychiatry
CAMH: Child and Adolescent Mental Health
ECP: Early Career Psychiatrists’
LAMIC: Low- and middle-income country
MD: Doctor of Medicine
NIMHANS: National Institute of Mental Health and Neuro Sciences
URL: Uniform Resource Locator
